# Interfacial, Electrical, and Band Alignment Characteristics of HfO_2_/Ge Stacks with In Situ-Formed SiO_2_ Interlayer by Plasma-Enhanced Atomic Layer Deposition

**DOI:** 10.1186/s11671-017-2083-z

**Published:** 2017-05-25

**Authors:** Yan-Qiang Cao, Bing Wu, Di Wu, Ai-Dong Li

**Affiliations:** National Laboratory of Solid State Microstructures and Department of Materials Science and Engineering, College of Engineering and Applied sciences, Collaborative Innovation Center of Advanced Microstructures, Nanjing University, Nanjing, 210093 People’s Republic of China

## Abstract

In situ-formed SiO_2_ was introduced into HfO_2_ gate dielectrics on Ge substrate as interlayer by plasma-enhanced atomic layer deposition (PEALD). The interfacial, electrical, and band alignment characteristics of the HfO_2_/SiO_2_ high-k gate dielectric stacks on Ge have been well investigated. It has been demonstrated that Si-O-Ge interlayer is formed on Ge surface during the in situ PEALD SiO_2_ deposition process. This interlayer shows fantastic thermal stability during annealing without obvious Hf-silicates formation. In addition, it can also suppress the GeO_2_ degradation. The electrical measurements show that capacitance equivalent thickness of 1.53 nm and a leakage current density of 2.1 × 10^−3^ A/cm^2^ at gate bias of V_fb_ + 1 V was obtained for the annealed sample. The conduction (valence) band offsets at the HfO_2_/SiO_2_/Ge interface with and without PDA are found to be 2.24 (2.69) and 2.48 (2.45) eV, respectively. These results indicate that in situ PEALD SiO_2_ may be a promising interfacial control layer for the realization of high-quality Ge-based transistor devices. Moreover, it can be demonstrated that PEALD is a much more powerful technology for ultrathin interfacial control layer deposition than MOCVD.

## Background

With the continuous scaling down of metal-oxide-semiconductor field-effect transistors (MOSFETs), Si-based MOSFET is approaching its physical and technical limitation. Alternative channel materials such as germanium (Ge) [1, 2] and III-V materials [3–5] have recently attracted a great deal of interest for high-performance logic device applications. Among them, Ge has the potential to replace silicon as the channel material in MOSFET because of its intrinsic higher hole carrier mobility [6]. However, direct deposition of high-k gate dielectrics on Ge substrates often causes high interface trap density (D_it_) and the unwanted formation of interfacial layer between Ge and high-k dielectrics layers [7]. Therefore, in order to achieve high-speed and low-power Ge-based MOSFETs, it is very important to achieve a high-quality high-*k*/Ge interface. Fortunately, a lot of methods have been reported to improve the quality of high-k/Ge interface [8], such as the introduction of SiO_2_ [9], Si [10], GeO_2_ [11], Al_2_O_3_ [12, 13], GeO_x_N_y_ [14, 15], and rare earth oxides [16, 17] as the interfacial control layer between Ge substrate and high-*k* gate dielectrics. In particular, the GeO_2_/Ge structure has superior interface properties, an extremely low interface state density (D_it_) of less than 1 × 10^11^ cm^−2^ eV^−1^ can be achieved [18]. However, GeO_2_ would decompose above 425 °C, and it is soluble in water. As a result, an unacceptable D_it_ is always revealed for the Ge-MOS capacitor (MOSCAP) [6]. Fortunately, Kita et al. reported that capping layer on GeO_2_ can suppress the GeO_2_ degradation; however, the selection of the material for the cap layer should be very crucial [19–21]. For example, Si or Y_2_O_3_ works more efficiently than HfO_2_ to retard the Ge-O desorption. These results indicate the importance of high-k materials or interfacial control layer selection to inhibit the GeO_2_ degradation. Nakashima et al. reported that a very thin SiO_2_/GeO_2_ bilayer by physical vapor deposition (PVD) is a promising interlayer layer for Ge passivation, a D_it_ of 4 × 10^11^ cm^-2^ eV^−1^ was achieved near the midgap [22, 23]. Li et al. introduced the SiO_2_ interlayer on Ge by metal-organic chemical vapor deposition (MOCVD), and SiO_2_ interlayer can effectively suppress Ge out-diffusion during HfO_2_ growth and subsequent post-deposition annealing process [9]. Therefore, SiO_2_ should be a wonderful interfacial control layer for Ge substrate. However, compared to PVD and MOCVD, PEALD can provide a much more uniform passivation layer, especially for ultrathin thickness. Hence, PEALD-formed SiO_2_ may be a promising interfacial control layer to achieve high-performance Ge-based transistor devices.

Herein, we introduced in situ PEALD-formed SiO_2_ into HfO_2_/Ge stacks as interfacial layer. The interfacial, electrical, and band alignment characteristics of ALD HfO_2_ films on n-type Ge substrates have been investigated carefully. The SiO_2_ was first deposited on the Ge substrates as interfacial control layer by PEALD. Then, HfO_2_ gate dielectric was in situ deposited by thermal ALD mode. Post-deposition annealing (PDA) at 500 °C for 60 s in N_2_ was performed for the HfO_2_/SiO_2_ high-k gate dielectric stacks on Ge. The X-ray photoelectron spectroscopy analyses reveal that Si-O-Ge interlayer and GeO_2_ layer is formed on the Ge surface during PEALD SiO_2_ deposition. This Si-O-Ge interlayer not only shows fantastic thermal stability, but also it can suppress the thermal decomposition of GeO_2_. Therefore, good electrical properties were achieved for the HfO_2_/Si-O-Ge/GeO_2_/Ge stacks. Compared to MOCVD SiO_2_ interlayer, in situ PEALD SiO_2_ exhibits much improved electrical properties. Therefore, PEALD is a much more powerful technology than MOCVD in the area of MOSFETs fabrication, especially for ultrathin interfacial control layer deposition.

## Methods

N-type Sb-doped Ge (100) with a resistivity of 0.2–0.3 Ω∙cm were used as substrates. The substrates were firstly cleaned by sonication in acetone, ethanol, isopropanol, and deionized water for 5 min, respectively. Then, a dilute HBr solution (H_2_O/HBr = 3:1) was used to etch the surface native oxides for 5 min. After wet chemical cleaning, the substrates were rinsed with deionized water and blown dry in pure N_2_. Subsequently, the substrates were immediately transferred to the PEALD (Picosun SUNALE^TM^ R-200) chamber. Before the high-*k* HfO_2_ films deposition, 10 cycles SiO_2_ film was deposited at 250 °C by PEALD as interlayer, where one cycle consisted of 1 s Si source injection, 10 s N_2_ purging, 13.5 s oxidant injection, and 4 s N_2_ purging. Tris-(dimethylamino)-silane (TDMAS) and O_2_ plasma were used as Si precursor and oxidant for SiO_2_ deposition, respectively. TDMAS was kept at room temperature. Pure O_2_ gas (99.999%) was used as O_2_ plasma source. The plasma power and O_2_ gas flow rate were 2500 W and 160 sccm, respectively. The growth rate of PEALD SiO_2_ was determined to be ~0.7 Å/cycle by ex situ spectroscopy ellipsometry. Then ~4 nm-thick HfO_2_ film was in situ deposited at 250 °C for 40 cycles by thermal ALD, where one cycle consisted of 0.1 s Hf source dosing, 4 s N_2_ purging, 0.1 s H_2_O dosing, and 4 s N_2_ purging. Tetrakis-(ethylmethylamino)-hafnium (TEMAH) and H_2_O were used as Hf precursor and oxidant for HfO_2_ deposition, respectively. TEMAH was evaporated at 150 °C and H_2_O was kept at room temperature. Pure N_2_ (99.999%) was used as carrier gas and purge gas. PDA was performed in N_2_ ambient at 500 °C for 60 s under atmospheric pressure using rapid thermal annealing.

The interfacial structures and chemical bonding of the films were investigated by ex situ X-ray photoelectron spectroscopy (XPS, Thermo Fisher K-Alpha) with standard Al Kα (1486.7 eV) X-ray source. XPS spectra were collected at a takeoff angle of 90°. The binding energy scale was calibrated using the Ge 3d_5/2_ peak at 29.4 eV. In addition, XPS spectra were fitted with Gaussian-Lorentzian (G-L) functions after smart-type background subtraction. Pt top electrodes of area 1.55 × 10^−4^ cm^2^ were deposited on the surface of HfO_2_ films using a shadow mask by sputtering method for electrical measurements. The capacitance-voltage (C-V) and leakage current density-voltage (J-V) characteristics were measured by a Keithley 4200 semiconductor analyzer system with a probe platform (Cascade summit 12000B-M).

## Results and Discussion

**Fig. 1:**
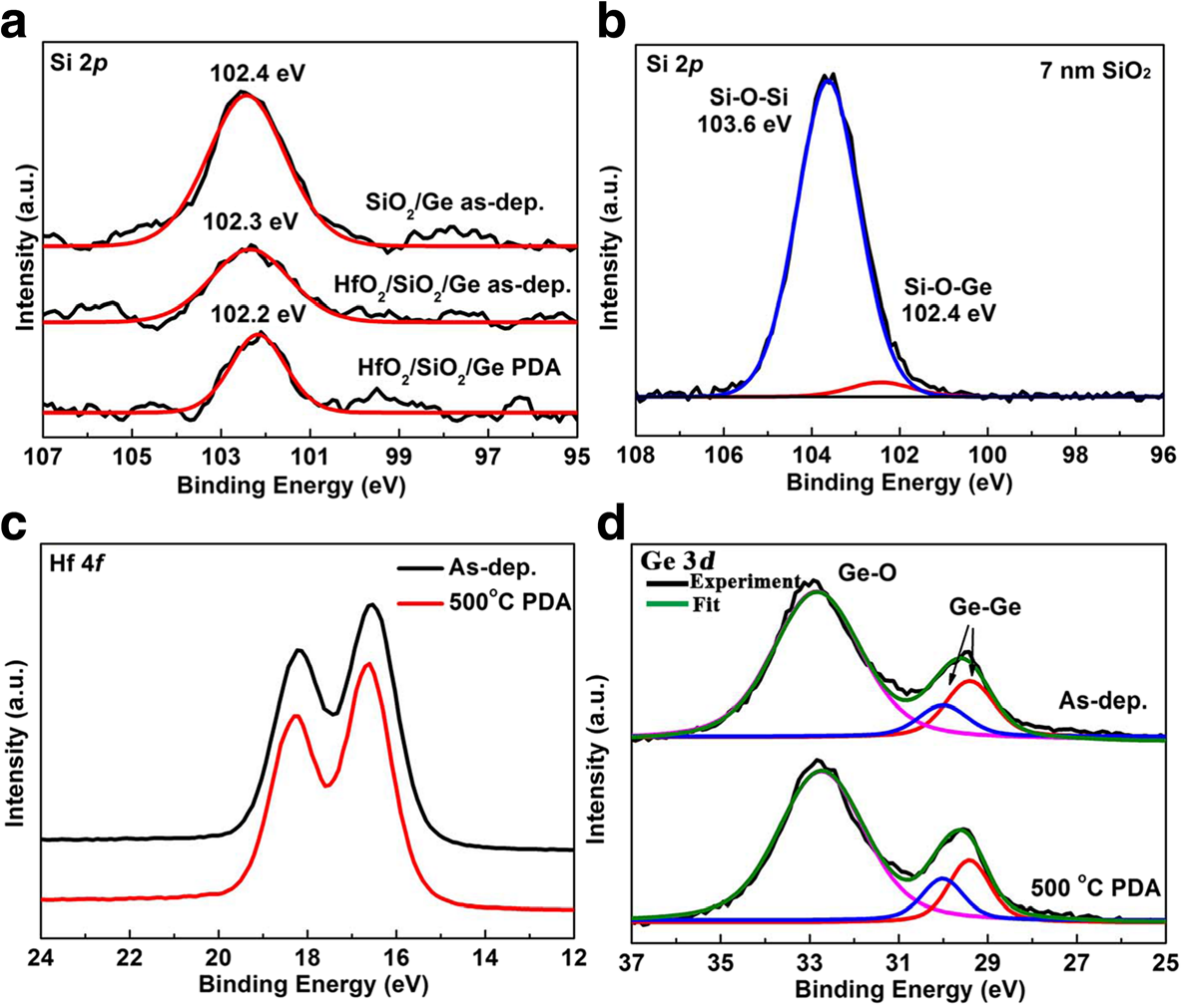
XPS spectra of SiO_2_/Ge and HfO_2_/SiO_2_/Ge structures. **a** Si 2p spectra of SiO_2_, as-deposited and annealed HfO_2_/SiO_2_ on Ge. **b** Si 2p spectra of thick SiO_2_(7 nm) on Ge. **c, d** Hf 4f and Ge 3d spectra of as-deposited and annealed HfO_2_/SiO_2_/Ge structures

For the thin PEALD SiO_2_ (~0.7 nm) on Ge, Si 2p exhibits a peak at 102.4 eV corresponding to Si-O bond (Fig. 1a), which is smaller than binding energy of ideal SiO_2_ [24]. Both silicon suboxide (SiO_x_) deposition and Si-O-Ge formation on Ge surface during PEALD process can cause the Si 2p shift to lower energy. Therefore, Si 2p spectrum of thick PEALD (~7 nm) on Ge was also performed. It can be found that it exhibits a main peak at 103.6 eV corresponding to ideal SiO_2_ bonding, as shown Fig. 1b. So, the silicon oxide deposited by PEALD here is ideal SiO_2_. However, besides the strong Si-O-Si peak, there is a weak peak located at ~102.4 eV, which should correspond to Si-O-Ge bonding on Ge surface. Therefore, it can be concluded that Si-O-Ge is formed on Ge surface in the initial PEALD SiO_2_ growth. After in situ 4 nm HfO_2_ deposition, the Si 2p peak intensity decreases without obvious chemical shift (102.3 eV), as shown in Fig. 1a. Furthermore, the Si 2*p* peak also exhibits no evident chemical shift (102.2 eV) after the 500 °C PDA in N_2_, suggesting the good thermal stability of the HfO_2_/SiO_2_ interface during HfO_2_ deposition and PDA process. In Hf 4f spectrum of as-deposited HfO_2_/SiO_2_ gate stacks (Fig. 1c), the doublet at 16.5 and 18.2 eV can be assigned to Hf 4f_7/2_ and Hf 4f_5/2_ peaks of HfO_2_ with the spin orbit splitting energy of 1.7 eV, consistent with the literature value of HfO_2_ [25]. After 500 °C PDA, the Hf 4f spectrum shows no obvious change with only 0.1 eV shift to higher energy. It implies that there are no evident Hf-silicates formed during PDA process. In Fig. 1d, the Ge 3d spectrum of as-deposited sample displays the doublet peaks at 29.4 and 30.0 eV, which can be assigned to the Ge 3d5/2 and Ge 3d3/2 peaks of Ge substrate with the spin orbit splitting energy of 0.6 eV. Except the signal of Ge substrate, there is a huge peak at 32.7 eV for Ge-O bonding. The Ge-O peak should be resulted from the formation of Ge-O-Si and GeO_2_. The GeO_2_ layer was formed by surface oxygen plasma oxidation during PEALD SiO_2_ deposition process. Therefore, the real fabricated structure here is HfO_2_/Si-O-Ge/GeO_2_/Ge stacks. Moreover, the Ge 3d spectrum shows no evident change after 500 °C PDA treatment, indicating the thermal stability of HfO_2_/Si-O-Ge/GeO_2_/Ge stacks without GeO_2_ degradation. It has been reported by Kita et al. that some capping layers on GeO_2_ could suppress the GeO_2_ decomposition, such as Si or La_2_O_3_ [19]. Therefore, the PEALD induced the Si-O-Ge interlayer here can also suppress the GeO_2_ decomposition. Based on above XPS analysis, it can be concluded that an ultrathin Si-O-Ge interlayer is formed on Ge surface. Moreover, this interlayer exhibits fantastic thermal stability without Hf-silicates formation, it can also inhibit the GeO_2_ degradation.

**Fig. 2:**
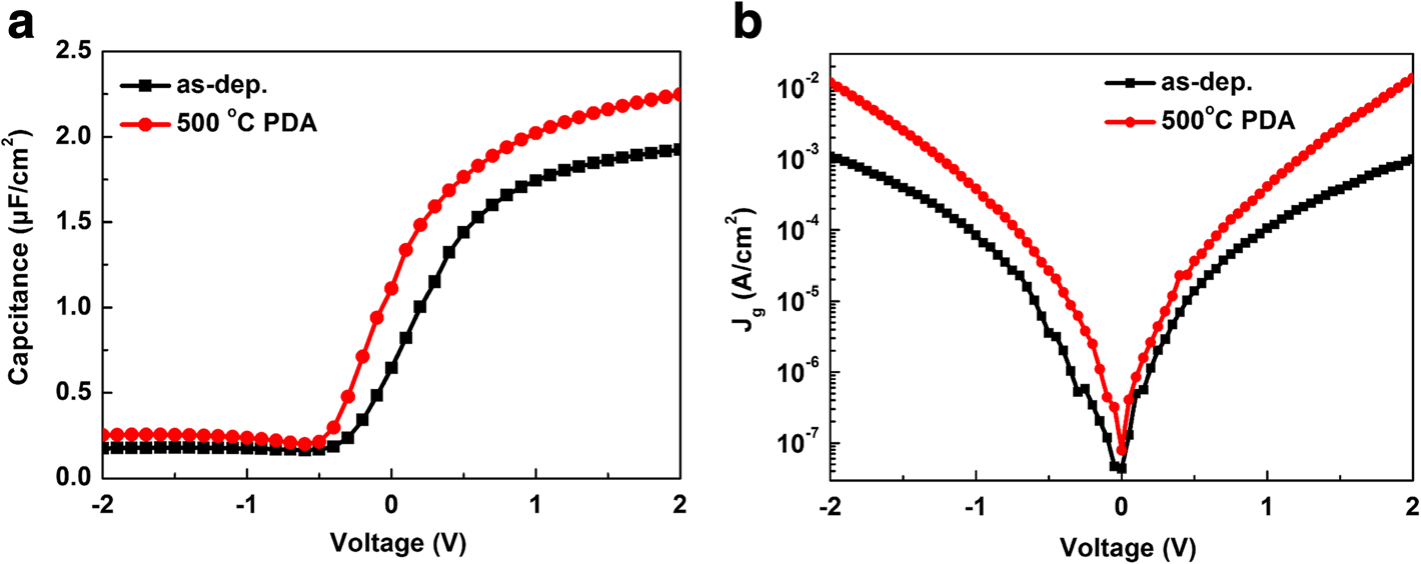
Electric characteristics of HfO_2_/SiO_2_ gate stacks on Ge substrates before and after 500 °C PDA. **a** High-frequency (1 M Hz) C-V curves. **b** J-V curves

Figure 2a plots the high-frequency (1 MHz) C-V curves of HfO_2_/SiO_2_ gate stacks on Ge before and after PDA. It can be found that flat band voltage (*V*_fb_) values of HfO_2_/SiO_2_/Ge before and after PDA are 0.42 and 0.27 V, respectively. The calculated ideal *V*_fb_ value is 0.55 V. The slightly negative *V*_fb_ shift indicates positive fixed charges, which may be induced by the oxygen vacancies in the dielectrics [26, 27]. During the inert atmosphere annealing process, more oxygen vacancies may be induced, resulting in a slightly negative *V*_fb_ shift. It has been demonstrated in many reported literatures that the GeO_2_ degradation during the annealing will cause the positive *V*_fb_ shift. The desorption process of Ge-O is believed to generate additional negative charges [28, 29]. Therefore, it can also be concluded that GeO_2_ decomposition is suppressed by Ge-O-Si interlayer from *V*_fb_ shift. The accumulation capacitance evidently increases from the original 1.92 to 2.25 μF/cm^2^ after PDA. The corresponding capacitance equivalent thickness (CET) values of the MOS capacitors can be calculated from the accumulation capacitances of the C-V curves using ε_0_ε_r_A/C_acc_ [30]. Therefore, a smaller CET of 1.53 nm is obtained after PDA compared to as-deposited sample of 1.80 nm. It can be ascribed to the fact that a denser and thinner high-k layer can be acquired after PDA process. Figure 2b shows the leakage current characteristics of HfO_2_/SiO_2_ films on Ge before and after PDA. At the bias voltage of *V*_fb_ + 1 V, the leakage current density is 2.1 × 10^−3^ A/cm^2^ and 2.2 × 10^−4^ A/cm^2^ for the sample before and after PDA, respectively. The increased leakage current density after PDA can be also attributed to the decrease of the gate dielectrics thickness.

**Fig. 3:**
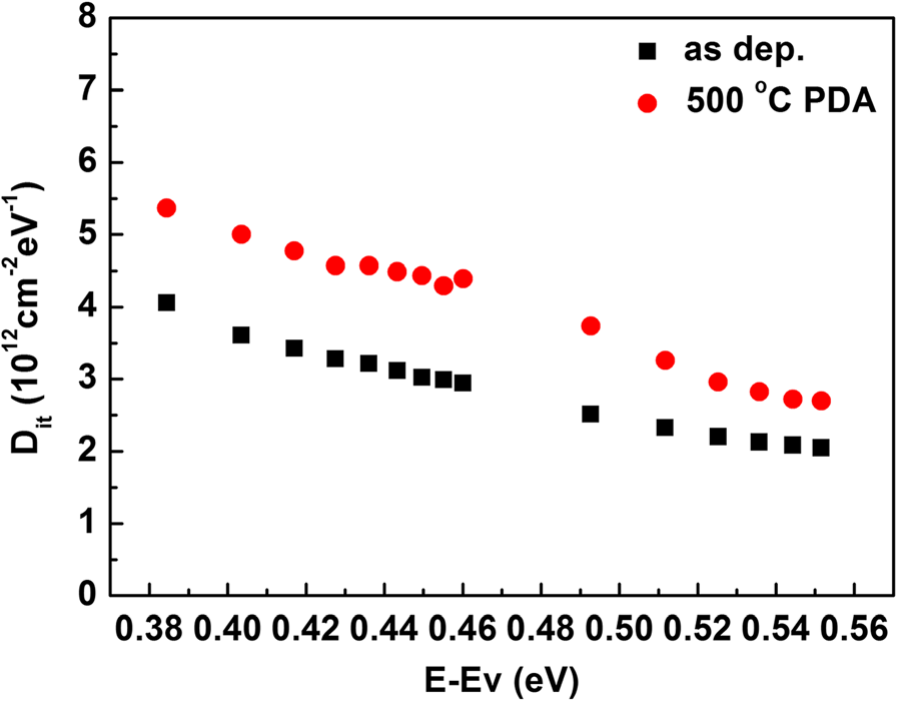
Distribution of D_it_ below E_c_ in the band gap at room temperature for Pt/HfO_2_/SiO_2_/Ge before and after 500 °C PDA

In order to examine the interface quality of HfO_2_/SiO_2_/Ge quantitatively, the interface state density (*D*_it_) was determined by the conductance method [31]. Figure 3 shows the distribution of *D*_it_ below E_c_ in the band gap extracted by the conductance method at room temperature for Pt/HfO_2_/SiO_2_/Ge before and after 500 °C PDA. The *D*_it_ can be roughly calculated from *D*_it_ = 2.5 × (*G*_p_/*w*)_max_/*A*q, where (*G*_p_/*w*)_max_ is the peak value of conductance-voltage characteristics, *f*(=*w*/2π) is the frequency, *A* is the electrode area, and *q* is the elemental charge. Therefore, *D*_it_ values of Pt/HfO_2_/SiO_2_/Ge structures without and with PDA are determined to be 4.05 × 10^12^ eV^−1^ cm^-2^ and 5.37 × 10^12^ eV^−1^ cm^−2^ at E-E_v_ = 0.38 eV, respectively. The lower *D*_it_ values of 2.03 × 10^12^ cm^−2^eV^−1^ and 2.67 × 10^12^ cm^−2^eV^-1^ near the bottom of conduction band are observed for the samples without and with PDA, respectively.

**Fig. 4:**
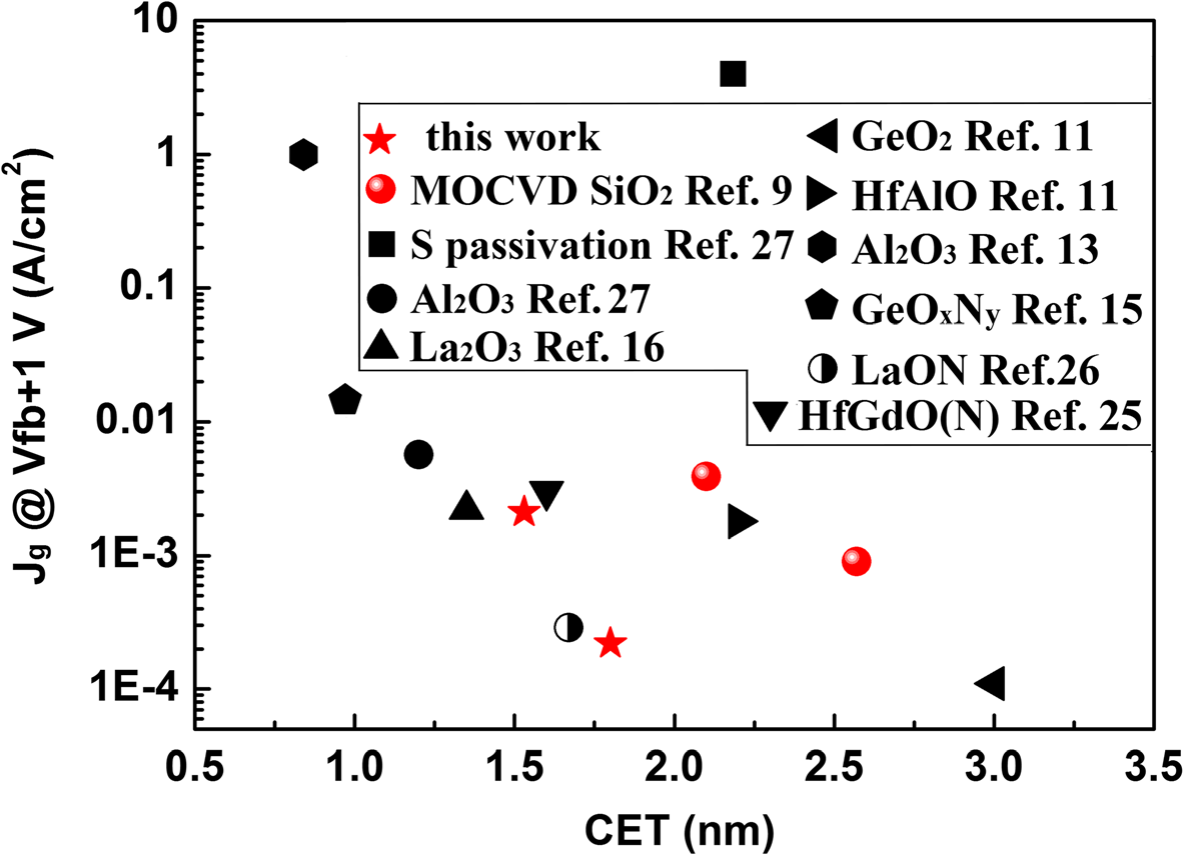
Leakage current density (Jg)-CET relationship for Ge-based MOS capacitors with different interfacial control layer

Figure 4 illustrates the leakage current density (*J*_g_)-CET relationship of Ge-based MOS capacitor with different interfacial control layer [32, 33]. Compared to the S-passivated Ge without interlayer reported by our previous work [34], the HfO_2_/SiO_2_/Ge in this work exhibits much improved properties with smaller CET (1.53 vs 2.18 nm), leakage current density (2.1 × 10^−3^ vs 3.1 A/cm^2^), and *D*_it_ (4.37 × 10^12^ vs 8.61 × 10^12^ eV^−1^ cm^−2^). It implies that in situ PEALD-formed SiO_2_ is a wonderful passivation layer for Ge. Moreover, compared to the ex situ-formed SiO_2_ interlayer by MOCVD [9], the sample with in situ PEALD-formed SiO_2_ interlayer in this work shows better electrical performance with both smaller CET (1.53 vs 1.75 nm) and leakage current density (2.1 vs 3.9 mA/cm^2^). It can be ascribed to the fact that SiO_2_ deposited by PEALD are more uniform than MOCVD especially for ultrathin thickness.

**Fig. 5:**
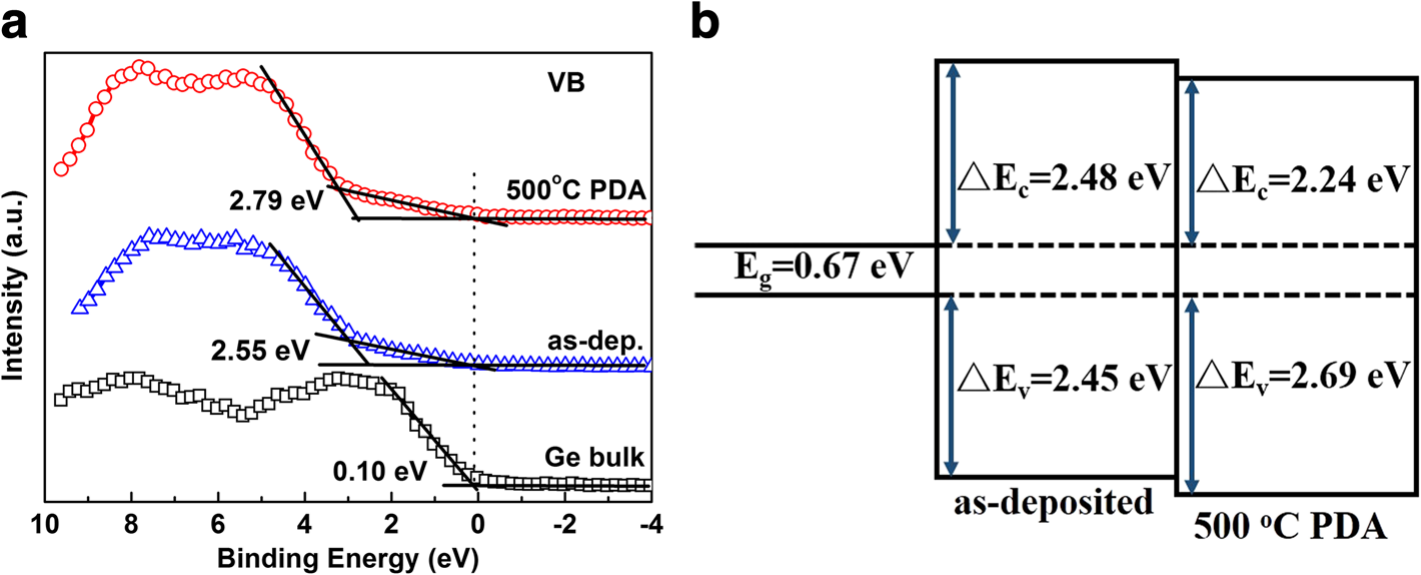
Band alignment of as-deposited and annealed HfO_2_/SiO_2_ film on Ge. **a**Valence-band spectra of the Ge substrate, as-deposited and annealed HfO_2_/SiO_2_ films. **b** Schematic of band alignment of as-deposited and annealed HfO_2_/SiO_2_ film on Ge

The band alignment at HfO_2_/SiO_2_/Ge interface was also determined by measuring the valence band offset ∆E_*v*_ (VBO) using XPS. The VBO values can be obtained based on the assumption that the energy difference between the core level and the valence band (VB) edge of the substrate remains constant with/without the deposition of dielectrics films [35]. Here, the Ge substrate was chosen as the reference to determine the VBO between gate dielectrics stack and Ge substrate. Figure 5a presents the VB spectra of the clean Ge substrate, as-deposited and annealed HfO_2_/SiO_2_/Ge stacks determined by linear extrapolation method, respectively. The VB edge of the clean Ge substrate has been determined to be 0.10 eV. And, the VB edges of as-deposited and annealed HfO_2_/SiO_2_ samples are found to be 2.55 and 2.79 eV, respectively. It can be noticed that there is a small tail in VB spectra for HfO_2_/SiO_2_/Ge stacks, which is corresponding to Ge substrate signal [36]. The leading edge of this weak tail is measured to be 0.10 eV and the same as the VB edge of Ge substrate. Therefore, the VBOs at the interface of HfO_2_/SiO_2_/Ge with and without PDA are estimated to be 2.69 and 2.45 eV, respectively. The conduction-band offset ∆*E*_*c*_ (CBO) can be obtained by subtracting the VBO and the bandgap of the substrate from the bandgap of HfO_2_:

$$ \varDelta {E}_c={E}_g\left({\mathrm{HfO}}_2\right) - {E}_g\left(\mathrm{Ge}\right) - \varDelta {E}_v, $$

where *E*_*g*_(HfO_2_) and *E*_*g*_(Ge) are the bandgap of HfO_2_ and Ge, respectively. The bandgaps of Ge and HfO_2_ are 0.67 and 5.6 eV, respectively. Therefore, the CBO values at the interface of HfO_2_/SiO_2_/Ge with and without PDA are estimated to be 2.24 and 2.48 eV, respectively. The CBO values are consistent with the previously reported data of 1.8–2.6 eV [37]. Figure 5b illustrates the corresponding band alignment of as-deposited and annealed HfO_2_/SiO_2_/Ge structures. Evidently, the HfO_2_/SiO_2_ high-k gate dielectric stacks on Ge exhibit large VBO and CBO values with huge barrier heights to inhibit leakage current.

## Conclusions

In summary, SiO_2_ interlayer was introduced into HfO_2_ gate dielectrics on n-Ge substrates successfully by in situ PEALD. We have investigated the interfacial, electrical properties, and band alignment of HfO_2_/SiO_2_/Ge MOS. It has been demonstrated that Ge-O-Si interlayer and GeO_2_ layer is formed on Ge surface during the in situ SiO_2_ deposition. This Ge-O-Si interlayer shows fantastic thermal stability during PDA without Hf-silicates formation. Moreover, Ge-O-Si interlayer can also inhibit the GeO_2_ degradation during annealing process. The HfO_2_/SiO_2_/Ge sample after PDA exhibits a CET value of 1.53 nm with low leakage current density of 2.1 × 10^−3^ A/cm^2^ at V_fb_ + 1 V. The VBO values at the HfO_2_/SiO_2_/Ge with and without PDA are determined to be 2.69 and 2.45 eV, and the CBO values to be 2.24 and 2.48 eV, respectively. Compared to the ex situ-formed SiO_2_ interlayer by MOCVD, the sample with in situ PEALD-formed SiO_2_ interlayer in this work shows improved electrical performance, ascribed to the fact that SiO_2_ deposited by PEALD are more uniform than MOCVD. Therefore, PEALD is a much more powerful technology for ultrathin interfacial control layer deposition than MOCVD.
